# Plant-Mediated RNAi for Controlling *Apolygus lucorum*

**DOI:** 10.3389/fpls.2019.00064

**Published:** 2019-02-06

**Authors:** Fangzhou Liu, Bin Yang, Aihong Zhang, Derong Ding, Guirong Wang

**Affiliations:** ^1^State Key Laboratory for Biology of Plant Diseases and Insect Pests, Institute of Plant Protection, Chinese Academy of Agricultural Sciences, Beijing, China; ^2^Hubei Insect Resources Utilization and Sustainable Pest Management Key Laboratory, College of Plant Science and Technology, Huazhong Agricultural University, Wuhan, China; ^3^Beijing DaBeiNong Biotechnology Co. Ltd., Beijing, China

**Keywords:** RNA interference, transgenic plants, non-Bt, lethal gene, *Apolygus lucorum*

## Abstract

The polyphagous mirid bug *Apolygus lucorum* (Heteroptera: Miridae) is a serious pest of agricultural crops in China, with more than 200 species of host plants including two very important crops, maize and soybean. Currently, prevention and control of *A. lucorum* rely mainly on chemical pesticides that cause environmental as well as health related problems. Plant-mediated RNAi has proven to offer great potential for pest control in the past decade. In this study, we screened and obtained seven candidate genes (*Aluc*β*-actin, AlucV-ATPase-A/D/E, AlucEif5A, AlucEcR-A, AlucIAP*) by injection-based RNAi which produced *A. lucorum* mortality rates of 46.01–82.32% at day 7 after injection. Among them, the plant-mediated RNAi of *AlucV-ATPase-E* was successfully introduced into transgenic maize and soybean, and the populations of *A. lucorum* were significantly decreased following feeding on the transgenic maize and soybean. These results are intended to provide helpful insight into the generation of bug-resistant plants through a plant-mediated RNAi strategy.

## Introduction

The green plant bug, *Apolygus lucorum*, is an important agricultural pest in China which has resulted in massive yield losses among more than 200 species of plants, including maize, soybean, cotton, and many other cash crops such as grape, apple, tea plant, and potato (Lu and Wu, 2008). The current methods for pest management of *A. lucorum* mainly rely on chemical pesticides, which often cause serious problems in environmental and safety issues. Thus, the development of other environmentally friendly methods is considered the first priority in controlling this pest. The commercial popularity of transgenic crops has undergone great development in the past two decades. Transgenic crops that produce *Bacillus thuringiensis* (Bt) insecticidal proteins successfully reduce the crop yield losses caused mainly by Lepidopteran pests; however, they have shown no toxicity toward Hemipteran and Hymenopteran insects. Therefore, developing new non-Bt methods to control pests from Hemiptera and Hymenoptera has become an imperative undertaking and a forward-thinking strategy to achieve control of these pests while minimizing risks to human and environmental health.

Plant-mediated RNA interference (RNAi), which involves introducing double-stranded RNAs (dsRNA) of critical genes from pests to crops, has become a new and successful approach to pest control. When feeding on the dsRNA, the pests will be adversely affected by the knockdown of critical genes which are essential to insect growth and development. In 2007, the first plant-mediated RNAi was applied on cotton, which expressed dsRNA specific to a cytochrome P450 gene (*CYP6AE14*), for the control the cotton bollworm, *Helicoverpa armigera* (Mao et al., 2007). Subsequently in the same year, several coleopteran species, most notably the western corn rootworm (WCR) *Diabrotica virgifera virgifera LeConte*, were significantly controlled by feeding transgenic corn plants that were engineered to express WCR *V-ATPase-A* dsRNA (Baum et al., 2007). Up to now, plant-mediated RNAi has been applied in several crops (including maize, rice, wheat, cotton, potato, tobacco, and many other important crops) to control numerous species among Lepidoptera, Coleoptera, and Hemiptera, including *H. armigera, Manduca* species, *D. v. virgifera, Myzus persicae, Sitobion avenae, Bemisia tabaci, Nilaparvata lugens, Adelphocoris suturalis, Leptinotarsa decemlineata*, and many others (Baum et al., 2007; Mao et al., 2007; Zha et al., 2011; Zhang et al., 2012; Mao and Zeng, 2013; Thakur et al., 2014; Xu et al., 2014; Luo et al., 2017; Spit et al., 2017). These studies have strongly demonstrated that plant-mediated RNAi is a feasible and powerful strategy for crop protection.

To develop the plant-mediated RNAi strategies, the first priority is to screen and acquire appropriate target genes, which have insecticidal effects on the target pests. In addition, these genes should also be safe to non-target organisms. Housekeeping genes are known to be crucial to the growth and development of insects, so they meet the first criterion for RNAi. Although housekeeping genes are usually relatively conservative among species, they may be applicable to RNAi if pest specific target fragments can be found through bioinformatics analysis. Housekeeping genes are essential for basic cellular functions throughout the whole life of a cell, and are expressed in all cells of an organism (Chretien et al., 1988). For example, *actins* are involved in many important cellular processes including muscle contraction, cell motility, cell division and others (Hanukoglu et al., 1983; Doherty and Mcmahon, 2008; Gunning et al., 2015). *Vacuolar-type H*^+^*-ATPase* (*V-ATPase*) is a highly conserved evolutionarily ancient enzyme. It is a complex composed of multiple subunits and plays a variety of roles crucial for the function of organelles such as endosomes, lysosomes, and secretory vesicles (Nelson et al., 2000; Fu et al., 2014). Besides housekeeping genes, many other genes also crucially affect the growth and development of insects, such as the genes related to energy metabolism and ecdysis. *Eukaryotic translation initiation factor 5A* (*Eif-5A*) is a cell protein that contains the unusual basic amino acid hypusine (Park, 2006). *Eif-5A* has proven to be involved in a variety of cellular processes including mRNA decay, cell cycle progression, apoptosis, translation elongation at polyproline sites, and stress responses (Hanauske-Abel et al., 1994; Zuk and Jacobson, 1998; Caraglia et al., 2003; Gosslau et al., 2009; Bian et al., 2017). In addition to the housekeeping genes, many other genes have proven to be essential for the physiological metabolism of insects. For example, *Ecdysone receptors* (*EcR*) are receptors for molting hormones, which play important roles in biological processes such as development, molting, metamorphosis, and reproduction in insects (Christiaens et al., 2010; Pieprzyk et al., 2014; Yu et al., 2014). Apoptosis, which is also called programmed cell death, is a cellular mechanism that is important in embryonic development, tissue homeostasis, and normal functioning of the immune system (Taylor et al., 2008). *Inhibitor of apoptosis* (*IAP*) proteins are key regulators of the innate antiviral response, which mediates a caspase-mediated apoptosis for limiting virus multiplication (Srinivasula and Ashwell, 2008; Orme and Meier, 2009). RNAi applications of the genes mentioned above have led to suppressed development and decreased populations in various insects to different extents, and they have indicated that these pest species-specific genes might be prioritized as ideal targets for plant-mediated RNAi targeting *A. lucorum* (Nelson et al., 2000; Katoh et al., 2004; Baum et al., 2007; Rumble and Duckett, 2008; Yu et al., 2014; Bian et al., 2017).

In this study, we screened and obtained seven target genes (including four housekeeping genes: β*-actin, V-ATPase-A/D/E*; and three other genes: *Eif-5A, EcR-A, IAP*) for controlling *A. lucorum* by injection-based RNAi. Furthermore, transgenic maize and soybean lines expressing *AlucV-ATPase-E* dsRNA were successfully constructed. Feeding bioassays under the green house conditions showed that feeding on transgenic crop lines could significantly suppress the development of *A. lucorum* and decrease their population. These results provide considerable examples of a plant-mediated RNAi approach to control pests in different crops and support the possibility of a new strategy for pest management in other pests and crops.

## Results

### Identification and Screening of Target Genes

**Figure 1:**
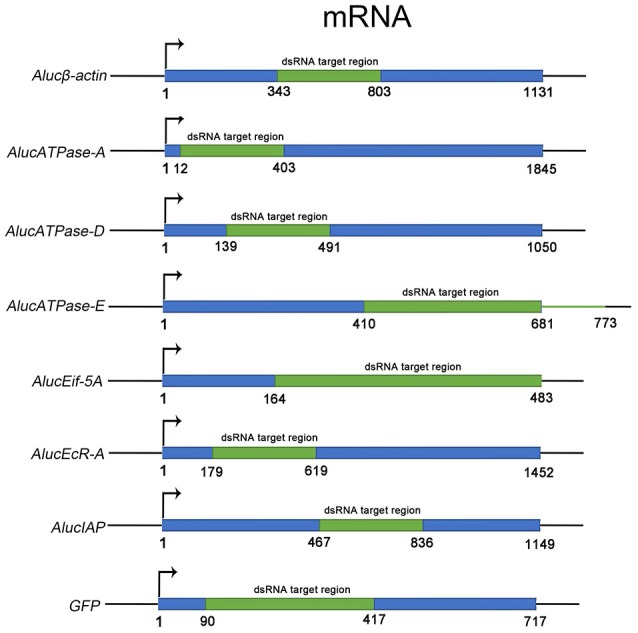
The fragments of dsRNA for each target gene. The ORF sequence of each target gene is shown in blue. Fragments in green indicate the regions for dsRNA. The numbers indicate the positions of the sequences from “ATG” to the last nucleotide base of the stop codon.

Through screening for the genes that are necessary for insect growth and development, we obtained several target genes in *A. lucorum*, including four housekeeping genes: β*-actin, V-ATPase-A/D/E*; and three other genes: *Eif-5A, EcR-A, IAP* (Figure 1). All the target genes which passed the safety-check by homologous examination against maize, soybean, and Human genomic or transcript libraries, were selected for RNAi development (Supplementary Data 1). Phylogenetic relationships between these genes in *A. lucorum* and other species are shown in Figure S1.

### Selection of the Best Injection Strategy

In the environment, pests should be controlled at an early instar. Because nymphs of *A. lucorum* at early instars are too soft for injection since they die too easily, as a compromise we selected the 3rd instar for injection. For the selection of the best injection strategy, two factors, position and amount for injection, were mainly considered in this study (Figure S2). For the control treatment 41.4 nL distilled water was injected into three positions. Mortality rates were significantly lower following injection at positions II and III (Figure S2B), namely 31.44 ± 3.53 and 31.54 ± 4.42%, respectively, compared to 51.66 ± 2.62% for position I treatment at day 7 after injection. Following injection of four volumes of distilled water at position II, Mortality rates were significantly lower for treatments which injected 27.6, 41.4, or 50.6 nL distilled water, while the rate was 60.97 ± 2.58% for the 101.2 nL treatment (Figure S2C). Because the mortality rate was slightly lower in the 27.6 and 41.4 nL treatments, considering that the RNAi should be more efficient from injection of more dsRNA, a 41.4 nL injection volume at position II was selected as the best injection strategy. The *GFP* gene was selected as control for RNAi, and injection of 41.4 nL ds*GFP* showed that the mortality rate was not significantly different from the DW-injected treatment, which was 29.56 ± 2.26% on day 7 after injection (Figure S2D).

### Lethal Gene Screening Using Injection-Based RNAi

**Figure 2:**
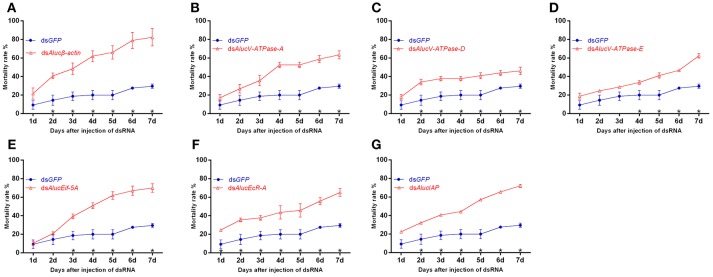
Mortality rates of injection-based RNAi for the target genes in *A. lucorum*. **(A)** *Aluc*β*-actin*; **(B–D)** *AlucV-ATPase-A/D/E*; **(E)** *AlucEif-5A*; **(F)** *AlucEcR-A*; **(G)** *AlucIAP*. Nymphs of the 3rd instar were used for this study. Error bars indicate statistical differences between three replicates (Mean ± SEM). The asterisks indicate the significant differences between RNAi treatments and control. The statistical analysis was done using Student's *t*-test (*P* < 0.05).

In total, seven dsRNAs including seven target genes and a *GFP* control were injected, respectively, into *A. lucorum*. Following injection of the dsRNA of these target genes, the bugs began to die from day 1. RNAi mediated high mortality from the injection of dsRNA of *Aluc*β*-actin* showed the highest mortality at day 7, with the value of 82.32 ± 9.39% (Figure 2A). Injected dsRNA of *AlucV-ATPase-A, AlucV-ATPase-E, AlucEif-5A, AlucEcR-A*, and *AlucIAP* resulted in mortality at day 7 of 63.39 ± 4.41, 62.34 ± 2.58, 69.94 ± 4.52, 65.05 ± 4.22, and 72.05 ± 2.90%, respectively (Figures 2B, D–G). The lowest mortality at day 7 was observed in the treatment of injected dsRNA of *AlucV-ATPase-D*, for which the mortality was 46.01 ± 4.00% (Figure 2C). Even in the lowest-mortality treatment, a significant difference between *AlucV-ATPase-D* and *GFP* was observed from day 2, and the mortality of injected ds*GFP* was 29.56 ± 2.26% at day 7. Actually, significant differences of mortality between targets and the control were observed at different days among the treatments. For example, a significant difference happened from day 1 in *AlucEcR-A* treatment while it only occurred after day 5 in *AlucV-ATPase-A* treatments. In addition, the death peaks were slightly different between treatments. Specifically, for *Aluc*β*-actin* and *AlucEcR-A* treatments the death peak occurred in day 2, for *AlucEif-5A* treatment it occurred in day 3, for *AlucV-ATPase-A* and *AlucV-ATPase-D* treatments it occurred in day 4, and for *AlucV-ATPase-E* treatment it occurred in day 7. In *AlucIAP* treatment, the mortality rate was equal day by day.

### Effect of RNAi on the Expression of Lethal Genes in *A. lucorum*

**Figure 3:**
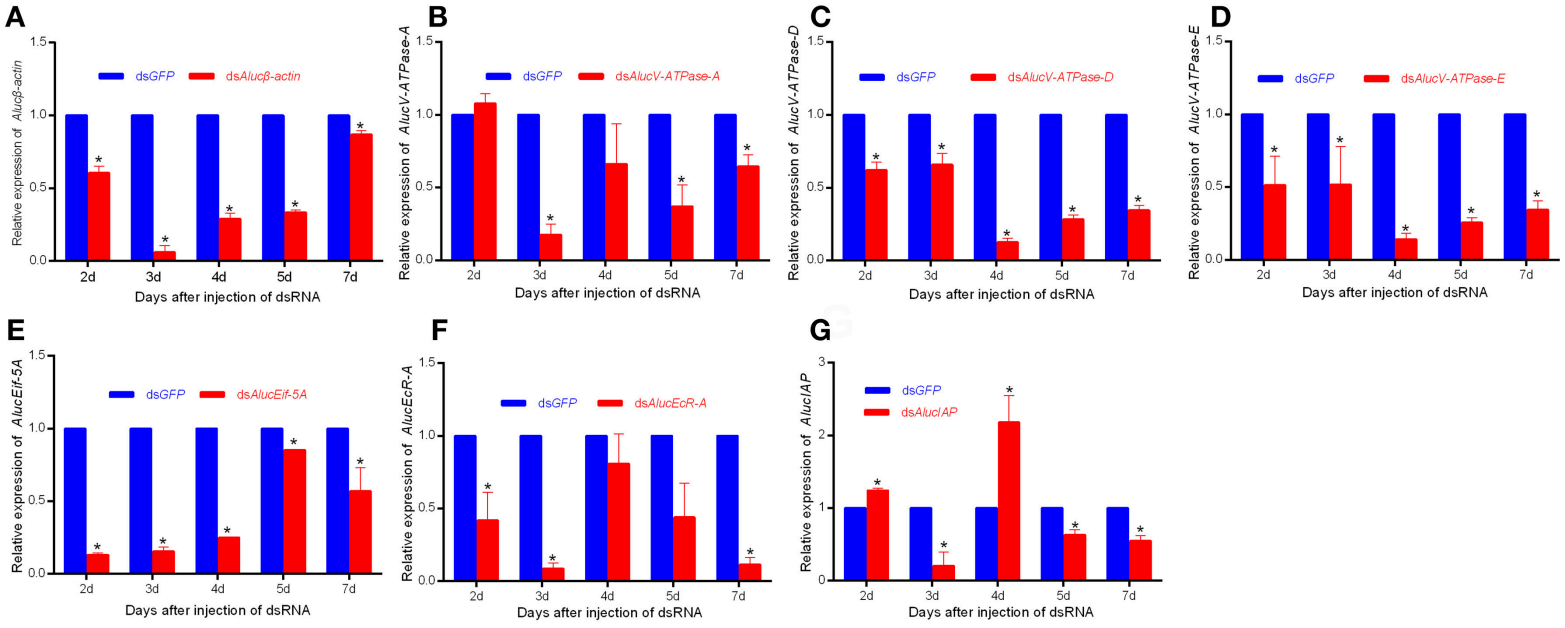
The relative expression level of each target gene for injection-based RNAi in *A. lucorum*. **(A)** *Aluc*β*-actin*; **(B–D)** *AlucV-ATPase-A/D/E*; **(E)** *AlucEif-5A*; **(F)** *AlucEcR-A*; **(G)** *AlucIAP*. Whole bodies of nymphs were collected from Day 2 to Day 7. *AlucGADPH* was used as reference control. Error bars indicate statistical differences between three replicates (Mean ± SEM). The target genes mRNA expression level in the ds*GFP* group was designated as one. The asterisks indicate the significant differences between RNAi treatments and control. The statistical analysis was done using Student's *t*-test (*P* < 0.05).

RNAi mediated inhibition of gene expression was examined by RT-qPCR. The relative mRNA expression levels of each target to *AlucGADPH* gene were significantly decreased compared with those in the *GFP* treatment. Expression levels of each gene from day 2 to day 7 are shown in Figure 3. For *Aluc*β*-actin* treatment, expression decreased 93.88 ± 7.81% in day 3, and recovered to only a 13.21% decrease in day 7. For *V-ATPase-A* treatment, expression decreased little in day 2, the maximum decrease of 82.41 ± 7.20% occurred in day 3, and it recovered to only a decrease of 35.54 ± 7.95% in day 7. For *AlucV-ATPase-D* and *AlucV-ATPase-E* treatments, the maximum decreases of expression levels occurred in day 4, with decreases of 82.41 ± 7.20 and 85.92±4.41%, respectively, which later recovered to 65.52 ± 3.49 and 65.52 ± 6.05% in day 7, respectively. Interestingly, in the *AlucEcR-A* and *AlucIAP* treatments, the relative expression levels reached maximum decreases in day 3, but recovered in day 4 and decreased again in days 5 and 7. This pattern also happened in day 7 for the *AlucEif5A* treatment.

### Construction and Molecular Screening of Transgenic Maize and Soybean

**Figure 4:**
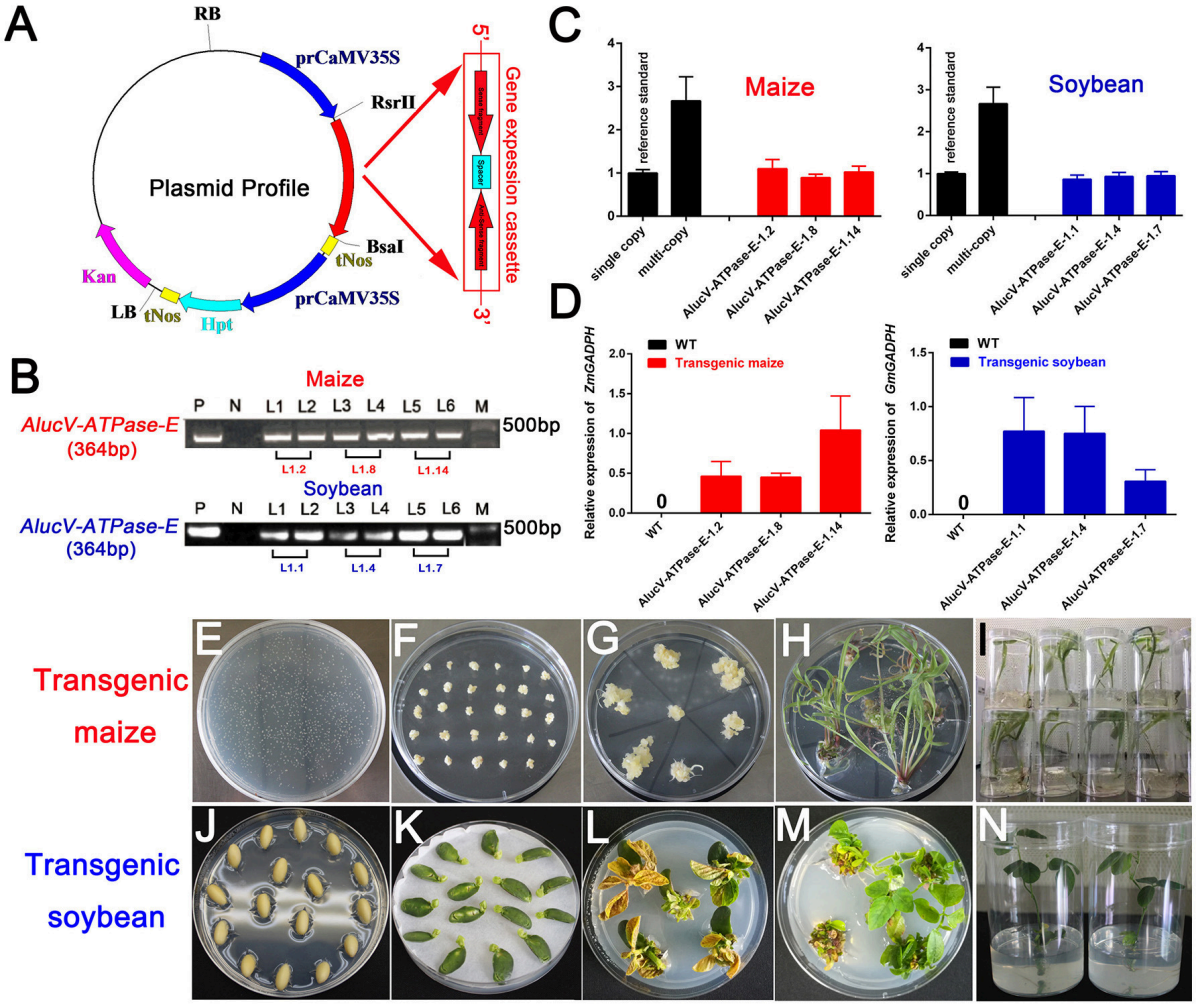
Construction and screening of the transgenic plants. **(A)** Construction of the plasmid vector for transformation. **(B)** PCR analysis for *V-ATPase-E* putative transgenic maize and soybean. M, marker; P, positive control; N, negative control. Numbers marked above the gel indicate the corresponding T0 transgenic plants. **(C)** TaqMan Real-time PCR assays for putative transgenic maize and soybean. **(D)** The relative expression of *AlucV-ATPase-E* dsRNAs in transgenic maize and soybean. *ZmGADPH* and *GmGADPH* were used as reference controls in maize and soybean, respectively. Three independent lines with three replicates of each line were used for the expression analysis. **(E–I)** Construction of transgenic maize by the *Agrobacterium*-mediated genetic transformation method. **(J–N)** Construction of transgenic soybean by the soy cotyledon node explants differentiation and regeneration techniques transformation method.

Among the target genes we screened using injection-based RNAi, *AlucV-ATPase-E* was selected for plant-mediated RNAi. The constructed vector is shown in Figure 4A. Transgenic maize and soybean were obtained by *Agrobacterium*-mediated genetic transformation of maize and soy cotyledon node explants through differentiation and regeneration techniques (Figures 4E–N). Site-specific integration was successfully induced to the genome of maize and soybean, and they were confirmed by RT-PCR and TaqMan Real-time PCR assays (Figures 4B–D). The obtained G0 transgenic plants, which contained single copies, were selected and planted in the greenhouse to obtain G1 offspring for subsequent studies. Expressions of dsRNA of *AlucV-ATPase-E* were confirmed in the G1 transgenic maize (three lines named 1.2, 1.8, and 1.14) and soybean (three lines named 1.1, 1.4, and 1.7) (Figure 4D).

### Plant-Dependent Lethal Effects for RNAi Among Different Genes

**Figure 5:**
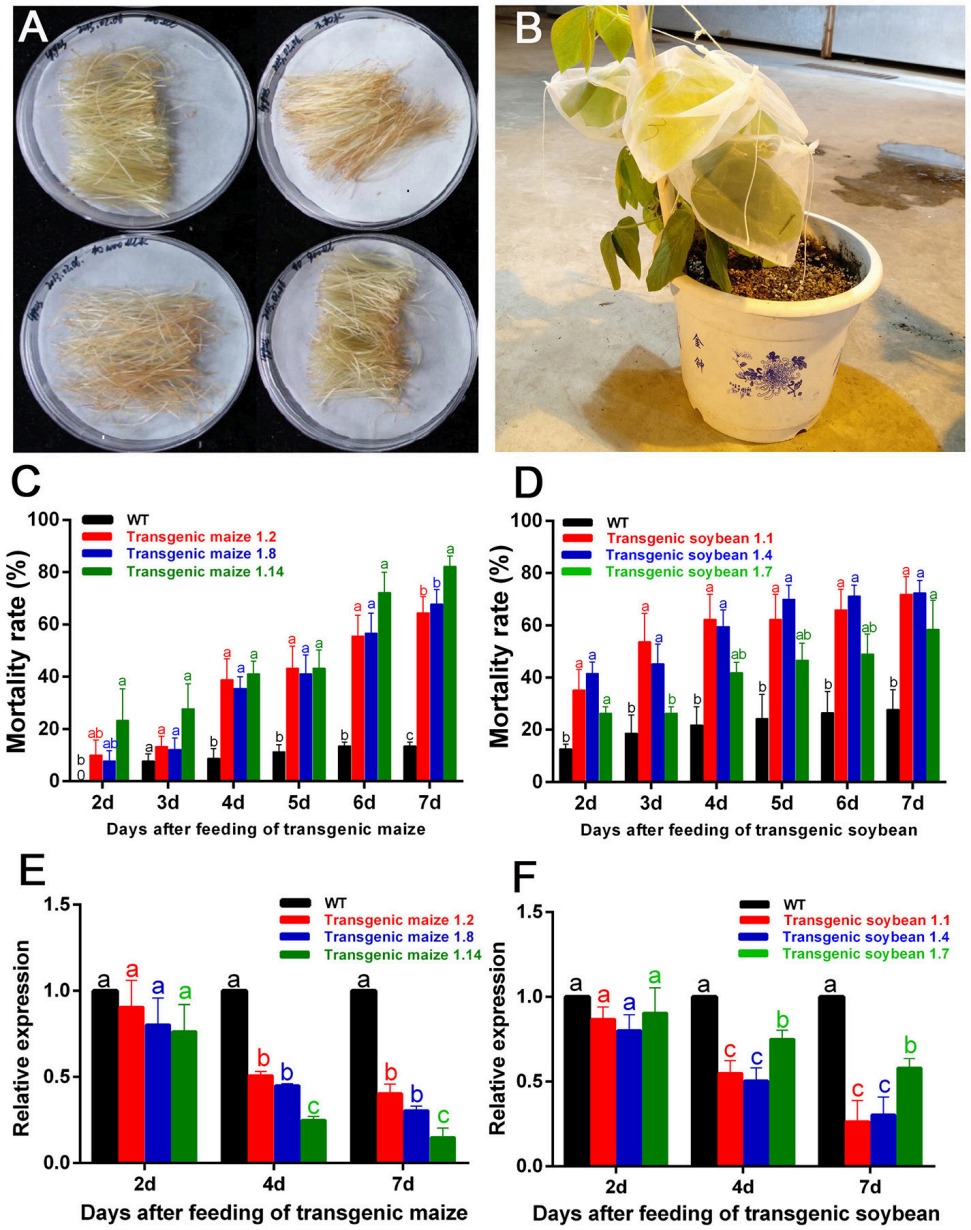
Feeding-bioassay of plant-mediated RNAi for *AlucA-ATPase-E* in *A. lucorum* using transgenic maize filaments and soybean leaves. About 30 individuals of the 3rd instar nymphs were reared on the fresh tissues of the wild-type and the positive transgenic lines. Three independent transgenic lines of maize filaments and soybean leaves from T1 transgenic plants were used for the assays. Three replicates were done for each trial. **(A,B)** The methods of feeding bioassay for *A. lucorum* with transgenic and negative control maize filament **(A)** and soybean leaves **(B)**. **(C,D)** Mortality rate of *A. lucorum* fed with transgenic maize filaments **(C)** and soybean leaves **(D)**. The mortality rates were calculated from Day 2 to Day 7. **(E,F)** Relative expression of target genes *AlucV-ATPase-E* after plant-mediated RNAi in *A. lucorum*. Whole bodies of *A. lucorum* were collected at day 2, day 4, and day 7 as indicated, *AlucGADPH* was used as reference control. The *AlucV-ATPase-E* mRNA expression level in the wild-type group was designated as one. Different letters (a, b, and c) indicate significantly different reductions relative to other treatments (*P* < 0.05, ANOVA, LSD, multiple comparisons test).

The filaments of G1 transgenic maize (three lines named 1.2, 1.8, and 1.14) and leaves of G1 transgenic soybean (three lines named 1.1, 1.4, and 1.7) were used to feed the nymphs of *A. lucorum* (Figures 5A,B). In the group fed transgenic maize of line 1.14, the mortality rates were significantly higher from Day 2 compared with the controls, and the mortalities in Day 7 were high, up to 80% (control: 13.50%) for plant-mediated RNAi of *AlucV-ATPase-E*. In the groups fed lines 1.2 and 1.8, the mortality rates were significantly higher from Day 3 compared with the controls, and the mortalities in Day 7 were high, up to 64.44 and 67.67%, respectively (Figure 5C). Expression levels of *AlucV-ATPase-E* genes were significantly reduced after feeding with all lines of transgenic maize (Figure 5E). In transgenic soybean lines, plant-mediated RNAi of *AlucV-ATPase-E* showed results similar to those in maize, mortality rates were significantly increased from Day 2, and the mortalities in Day 7 were 71.79, 72.47, and 58.48%, for lines 1.1, 1.4, and 1.7, respectively (control: 29.76%) (Figure 5D). Expression levels of *AlucV-ATPase-E* were significantly reduced after feeding with all lines of transgenic soybean (Figure 5F).

## Discussion

RNAi technology is one of the greatest inventions of the last century and it has had a tremendous impact on the molecular science of biology. It is a powerful tool for functional study by suppressing the expression of target genes both *in vitro* and *in vivo*. The loss-of-function strategies have made a path to screen RNAi-induced lethal genes for pest control in an environmentally friendly way. As a novel and safe strategy for pest management, plant-mediated RNAi has been applied in many species for more than 10 years since the first application in 2007 (Huvenne and Smagghe, 2010). In this study, we obtained two plant-mediated RNAi crops that generated high mortality rates, up to about 70%, in the feeding bioassays. This rate is comparable to previous studies where the mortality rates mostly ranged from 20% to about 70% (Christiaens and Smagghe, 2014). Most plant-mediated RNAi applications are still at the research stage. However, an exciting milestone was the development of a new transgenic maize variety, MON87411, expressing one dsRNA of *Sucrose non-fermenting 7* (*DvSnf7*) with *three Crystal* (*Cry*) genes for controlling *D. v. virgifera*, which was approved for production as commercial products by the U.S. Environmental Protection Agency (EPA) in 2017 (U. S. EPA, 2017). It is the first plant-mediated RNAi product that acquired permission from the government, which elevates the potential for commercialization of plant-mediated RNAi worldwide.

Pests with piercing-sucking mouthparts, especially Hemiptera, have caused massive crop yield losses not only by their herbivorous nature but also because they are important vectors of devastating diseases and viruses (Christiaens and Smagghe, 2014). Most of them have already developed high levels of resistance to the conventional and modern insecticide groups (Ma et al., 2007; Luo et al., 2010). It is therefore promising to find that RNAi works very well among pests with piercing-sucking mouthparts because they are also highly sensitive to injection- and feeding-mediated RNAi. However, plant-mediated RNAi may not affect them since these pests are feeding on plant phloem sap, where dsRNA is not expressed. Fortunately, *A. lucorum* has a special way of using their mouthparts: the bugs first use their stylets to lacerate the plant cells, simultaneously secreting watery saliva into the ruptured cellular matter, and then they ingest the mixed pulp (Backus et al., 2007). This “lacerating then sucking” mechanism for feeding offers a route by which the dsRNA could be taken in, because the plant cells express high levels of dsRNA (Wheeler, 2001). Based on this theory, we were determined to construct the transgenic maize and soybean that express dsRNA to detect whether plant-mediated RNAi is feasible for management of *A. lucorum*. Our results demonstrated a successful application of plant-mediated RNAi in Hemipteran insects, as feeding on transgenic plants significantly suppressed the development and decreased the population of *A. lucorum* in both maize and soybean—although the mortality in soybean (67.72%, average of three lines, day 7) was lower than that in maize (72.43%, average of three lines, day 7).

Issues of safety to non-target organisms should be taken into careful consideration when plant-mediated RNAi are applied. In addition to high lethal effects toward pests, preventing off-target effects is another serious point for the design of dsRNA. Fragments of a target gene in pests should be less conserved compared to those of the host plant, other natural enemies and even human beings. In this case, housekeeping genes may not be the first choice because they are usually highly conserved among species. However, we found some fragments in housekeeping genes that were less conserved. In this study, *Aluc*β*-actin* and *AlucV-ATPase-A/D* were excluded from the construction of transgenic crops because the target fragment sequences contained at least 15 sections (19–20 bp) which matched the genomes of maize, soybean or human. However, this study is the first to develop plant-mediated RNAi for controlling *A. lucorum*, and we selected long fragments (about 400 bp) as the targets for RNAi. Even with this condition, *AlucV-ATPase-E* showed less similarity to sequences from maize, soybean, human, and some other insects (Supplementary Data 1). To reduce the off-target effects and make the target sequences more specific to the pests, shorter fragments or siRNA will be selected in later research.

In the screening of target genes by injection-based RNAi, a special phenomenon was observed. In treatments with RNAi for *AlucV-ATPase-A, AlucEif-5A, AlucEcR-A*, and *AlucIAP*, gene expression was firstly suppressed, subsequently rebounded to a high expression level, and then became suppressed again. This was especially pronounced in the case to silence *AlucIAP*, in which the expression level was two times higher than that of the control in day 4. This kind of result has also appeared in many other studies (Li et al., 2011). The reason for its occurrence is still not clear, but it might possibly result from some stress reaction occurring for the adventive stimulation, then the suppressed gene being restored to the normal expression level by upstream genes, but the rebound effect subsequently making the gene expression higher than that in wildtype.

Above all, plant-mediated RNAi is a potential technique for *A. lucorum* management, but several general points should be taken into consideration: (1) Silencing the target gene should induce significant negative effects for insect development or other behaviors, such as mating and feeding. (2) Target fragments selected should be less conserved compared to crops and other organisms such as beneficial insects and human beings, which would make it applicable in the crops and reduce the possibility for biosecurity problems. (3) Silencing one target gene may display similar lethal effects among transgenic plants, but it nseeds to be further identified in a wide range of crops.

## Conclusions

In this study, we screened and obtained seven candidate genes which are important for insect development and growth using injection-based RNAi. Among them, plant-mediated RNAi of *AlucV-ATPase-E* was successfully constructed into transgenic maize and soybean. Silencing *AlucV-ATPase-E* significantly suppressed the development and decreased the population of *A. lucorum* in both transgenic maize and soybean, indicating that it is an effective target for controlling *A. lucorum*. Our results demonstrate a potential target gene which might be commercially applicable for plant-mediated RNAi in pest management of *A. lucorum*, and reveal that plant-mediated RNAi might also be a feasible method for controlling other pests.

## Materials and Methods

### Insect Rearing

*A. lucorum* was maintained in the laboratory conditions at the Institute of Plant Protection, Chinese Academy of Agricultural Sciences, Beijing, China. Insects were reared at 28 ± 1°C and 60 ± 5% relative humidity (RH), under a 14:10 light/dark cycle. The nymphs were synchronized by collection immediately after hatching and fed on maize (*Zea mays*) and soybean (*Glycine max*). The insects were reared in a plastic box (20 × 15 × 8 cm) covered with a gauze at a density of 100–150 individuals per box. Nymphs of 3rd instar were collected for injection at about 96–100 h after hatching.

### Identification and Screening of Target Genes

All the target genes were identified by homology screening from other species by local BLAST using the next generation sequencing data of *A. lucorum* (Cao et al., 2016). Among them, *Aluc*β*-actin* (KU188517.1), *AlucEcR* (KM401656.1), and *AlucIAP* (KP100065.1) were already deposited in the National Center for Biotechnology Information (NCBI) Database. *GFP* (AccNo. U76561) was selected as a control (Nunes et al., 2013). Homologous sequences were downloaded from the NCBI database including *V-ATPase-A* (XM_018110614.1), *V-ATPase-D* (XM_011202115.2), *V-ATPase-E* (XM_011193708.1), and *Eif-5A* (DQ202521.1) from other insects. Blastn with e-value (0.00001) was used for the screening. Phylogenetic relationships were deduced by the neighbor-joining method using Molecular Evolutionary Genetics Analysis (MEGA) software 6 (Tamura et al., 2013). The screened target genes were then blasted against the maize, soybean and Human genomic or transcript data on NCBI (Supplementary Data 1). The sequences of target genes should not contain more than 19 consecutive bases homologous to the Human genomic or transcript sequences.

### Total RNA Extraction and cDNA Synthesis

Total RNA was extracted using TRIzol Reagent (Invitrogen, Carlsbad, CA, United States) and then cDNA was synthesized using Transcript One-Step gDNA Removal and cDNA Synthesis SuperMix Kit (TransGen Biotech, China) according to the manufacturer's instructions. Products were quantified using NanoDrop2000 (NanoDrop, Wilmington, DE, United States) and quality assessed by agarose gel electrophoresis.

### Double-Stranded RNA Synthesis

Primers for amplifying the target regions were designed using Primer Premier 5 (Lalitha, 2000). The dsRNAs were constructed in lengths of 350–450 bp, amplified using designed primers which fused a T7 sequence (5′-TAATACGACTCACTATAGGG-3′) to the 5′ ends of both sense and antisense, and cloned into PGM-T vector (TIANGEN, Beijing, China) (Table S1). Amplified products were purified using a QIAquickTM PCR purification kit (Qiagen, Hilden, Germany), and then the dsRNAs constructs were synthesized using a T7 RiboMAX Express RNAi System (Promega, Madison, Unites States) following the manufacturer's instructions. The resulting dsRNAs were purified by phenol/chloroform extraction methods and dissolved in nuclease-free water. The concentrations of dsRNAs were measured using NanoDrop2000 (NanoDrop, Wilmington, DE, United States), and verified in 1.0% agarose gels. Finally, the dsRNAs were diluted to 10 μg/μl, split into 4 μl aliquots and kept at −80°C for later use.

### RNA Interference Using Microinjection

The 3rd instar nymphs were anesthetized with CO_2_ (PCO_2_ = 1 mPa, 40 s) and placed on a 1.5% agarose plate with the ventral surface facing up. Four volumes (27.6, 41.4, 50.6, and 101.2 nL) of distilled water (DW) were injected into three different positions using NANOlatter 2000 (WPI, United States) (Figure S2). Injected nymphs were maintained in Petri dishes with fresh corn kernels, and the mortality rate of each treatment was recorded from day 1 to day 7 after injection. At least 50 nymphs were injected for each injection treatment, and three technical replicates were set for examining the results. The mortality rate of each treatment was analyzed for selection of the best injection strategy. Based on these results, dsRNAs of target genes were injected using the best injection strategy (41.4 nL at injection position II), and the mortality rates were recorded from day 1 to day 7 after injection.

### Quantitative Reverse Transcription PCR

The relative expression levels of the target genes after injection were validated by quantitative reverse transcription PCR (RT-qPCR). Nine nymphs from three biological replicates were collected randomly from 2 to 7 days after injection, total RNA from whole bodies of each replicate was extracted as a template following the description mentioned above. Quantitative PCR (RT-qPCR) was performed and analyzed with ABI Prism 7500 Fast Detection System (Applied Biosystems, Carlsbad, CA, United States). *Nicotinamide adenine dinucleotide phosphate oxidase* (*AlucGADPH*) gene was included in the analysis as an endogenous control to normalize the target gene expression. Primers for each gene were designed to amplify an approximately 150 bp-long fragment at the 3′ end of the ORF of each gene (Table S1). Relative expression levels were analyzed by the 2^−ΔΔ*CT*^ method (Livak and Schmittgen, 2001). Three biological replicates, each with three technical replicates, were averaged.

### Constructions of Transgenic Maize and Soybean

The target fragments for RNAi were selected from the coding DNA sequences (CDS) of *AlucV-ATPase-E* gene (Figure 1). The vectors for transgenic maize and soybean were constructed from several domains including a *CaMV35S* promotor, a gene expression cassette, and a tNos terminator, followed by another *CaMV35S* promotor connected with an Hpt marker gene and tNos terminator. The gene expression cassette contained a target gene sense sequence (SS), a PDK intron and a target gene antisense sequence (AS) linked together via restriction enzyme sites as RsrII-SS-Hind III-PDK-Bsa I-AS-Xho I. All the vectors were constructed according to the manufacturer's recommendations. The transgenic maize and soybean were constructed by *Agrobacterium tumefaciens*-mediated transformation and soy cotyledon node explant differentiation and regeneration techniques, respectively (Zhao et al., 2002; Paz et al., 2004).

### Molecular Analysis for Transgenic Maize and Soybean

Transgenic plants were screened using RT-PCR, RT-qPCR, and TaqMan Real-time PCR assays. Among them, TaqMan Real-time PCR is used to detect the copy numbers of target fragments in transgenic plants by contrast the single copy reference standard. RT-qPCR is used to estimate the expression levels of dsRNA in the transgenic plants. Genomic DNA was extracted from maize filaments and soybean young leaves for putative transgenic candidates using a Plant Genomic DNA Kit (Tiangen Biotech, Beijing, China). The genomic DNA of established single-/multi-copy maize and soybean were selected as reference controls. Vectors for transgenic maize/soybean were used as positive controls, while the wild-type maize/soybean were used as negative controls. To examine the expression of dsRNA in maize and soybean, RT-qPCR was carried out following the methods described above. Filaments of maize and leaves of soybean were used for RNA isolation. *ZmGADPH* and *GmGADPH* were selected as reference controls in maize and soybean, respectively (Bansal et al., 2015; Jue et al., 2015). From more than 20 positive lines, three were used for the expression analysis, with three replicates of each line. All the primers mentioned above are shown in Table S1.

### Feeding-Bioassay for *A. lucorum* Using Transgenic Plants

The effects of plant-mediated RNAi were carried out by feeding-based bioassay. Three independent transgenic lines of maize filaments and soybean leaves from T1 transgenic plants and wild-type plants were used for the assays. Thirty individuals of 3rd-instar nymphs were reared with the selected tissues and their mortality rates were calculated for 7 days. Three replicates were done for each trial. In addition, RT-qPCR was carried out to examine the expression of the target genes in *A. lucorum*. Whole bodies of *A. lucorum* were collected at day 2, day 4, and day 7. The Student's *t*-test was used to perform the statistical analyses of the data.

## Author Contributions

BY and GW: designed the research. FL, AZ, and DD: performed experiments. BY and FL: analyzed the data and wrote the manuscript.

### Conflict of Interest Statement

The authors declare that the research was conducted in the absence of any commercial or financial relationships that could be construed as a potential conflict of interest.

## Supplementary Material

Figure S1 Phylogenetic tree of **(A)** *Aluc*β*-actin*; **(B–D)** *AlucV-ATPase-A/D/E*; **(E)** *AlucEif-5A*; **(F)** *AlucEcR-A*; **(G)** *AlucIAP*.

Figure S2 The sites for microinjection and exploring optimum injection parameters for the 3rd instar nymphs of *A. lucorum*. **(A)** Position I: the conjunctives between the edges of the conjunctive prothorax and mesothorax; Position II: the conjunctives between the edges of the conjunctive mesothorax and metathorax; Position III: the edge of the conjunctive between the second and third abdominal segments. **(B)** Mortality rate induced by microinjection at different positions with 41.4 nL of water. **(C)** Mortality rate induced by microinjection at position II with different volumes of water. **(D)** Mortality rate induced by microinjection with 41.4 nL of water and ds*GFP*. All error bars indicate the SEM of the mean, as determined from three independent replicates. Bars labeled with asterisks differed significantly between the treatments on the same day, as determined using an LSD pair-wise comparison of means. The level of significance for results was set at *P* < 0.05.

Table S1 Primers used in this study.

Supplementary Data 1 The alignments of target genes. Sequences comparisons were performed using DNAman software. Target genes were alignment with the homologous gene from the representatives species of Diptera (*Drosophila melanogaster*), Lepidoptera (*Helicoverpa armigera*), Coleoptera(*Tribolium castaneum*), Hymenopteran (*Apis mellifera*), Hemiptera (*Acyrthosiphon pisum*) and the species of maize, soybean, and humans. dsRNAs design area marked with blue box.
