# Supplementary figures and images for: Plant-Mediated RNAi for Controlling Apolygus lucorum
Source: Front Plant Sci. 2019 Feb 6;10:64. doi: 10.3389/fpls.2019.00064 (PMC6374644; doi:10.3389/fpls.2019.00064)

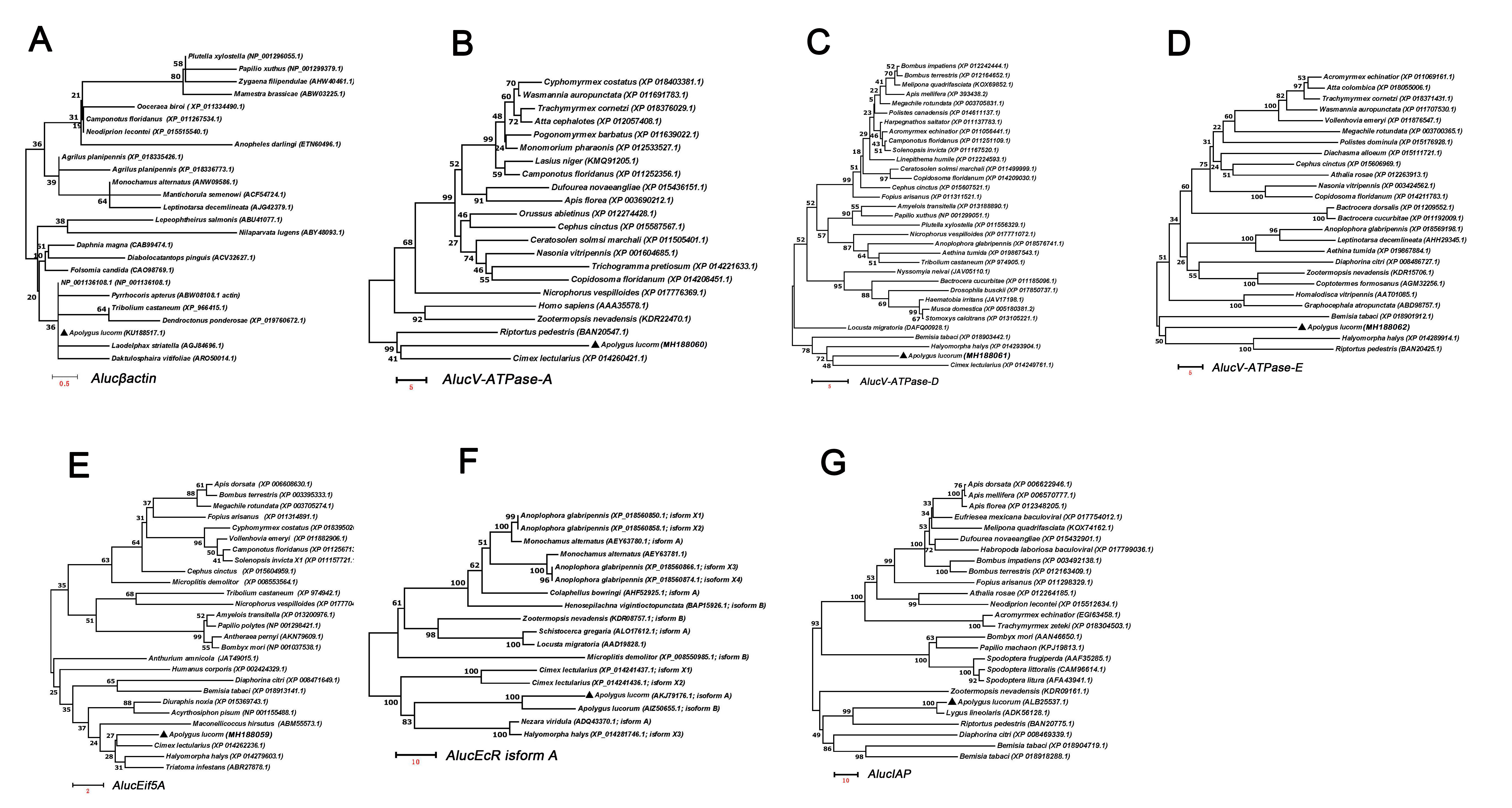

Supplement: Figure S1 — Phylogenetic tree of (A) Alucβ-actin; (B–D) AlucV-ATPase-A/D/E; (E) AlucEif-5A; (F) AlucEcR-A; (G) AlucIAP. [file Image_1.JPEG]

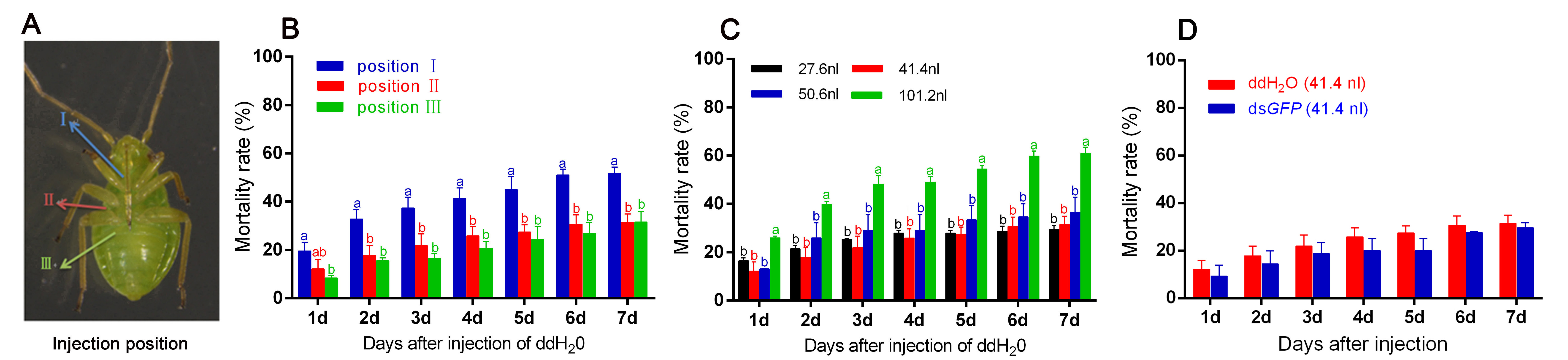

Supplement: Figure S2 — The sites for microinjection and exploring optimum injection parameters for the 3rd instar nymphs of A. lucorum. (A) Position I: the conjunctives between the edges of the conjunctive prothorax and mesothorax; Position II: the conjunctives between the edges of the conjunctive mesothorax and metathorax; Position III: the edge of the conjunctive between the second and third abdominal segments. (B) Mortality rate induced by microinjection at different positions with 41.4 nL of water. (C) Mortality rate induced by microinjection at position II with different volumes of water. (D) Mortality rate induced by microinjection with 41.4 nL of water and dsGFP. All error bars indicate the SEM of the mean, as determined from three independent replicates. Bars labeled with asterisks differed significantly between the treatments on the same day, as determined using an LSD pair-wise comparison of means. The level of significance for results was set at P < 0.05. [file Image_2.jpeg]
