# Supplementary material for: Plant-Mediated RNAi for Controlling Apolygus lucorum
Source: Front Plant Sci. 2019 Feb 6;10:64. doi: 10.3389/fpls.2019.00064 (PMC6374644; doi:10.3389/fpls.2019.00064)
Supplement: Table S1 — Primers used in this study. [file Table_1.DOCX]

**Table S1: Primers used in this study.**

| **Gene name** | **Primers synthesized for different usage** | |
| --- | --- | --- |
| *Alucβ-actin* | Primers for EST cloning | **F: 5’ ATGTG(CT)GACGA(ATC)GA(AT)GTTGC 3’** |
|  |  | **R: 5’ TTAGAA(TG)CA(TC)TT(CAC)CTGTG 3’** |
|  | Primers for RT-qPCR | **F: ACCGCTGAAAGGGAAATCG** |
|  |  | **R:ATGACCTGACCGTCGGGAA** |
|  | Primers for ds*Alucβ-actin* synthesis | **F: TAATACGACTCACTATAGGGGCCAACAGGGAGAAGATG** |
|  |  | **R: TAATACGACTCACTATAGGGAGGAAGGAAGGCTGGAAT** |
| *AlucV-ATPase-A* | primers for EST cloning | **F: TTTACCCAGAATCCGTGAT** |
|  |  | **R: ATGGAGTGAAGTCCCAAGC** |
|  | Primers for RT-qPCR | **F: GTTGAAATTGATGGCGTTACTGAG** |
|  |  | **R: CGGAAGTATTCGGACAAGGTGA** |
|  | Primers for ds*AlucV-ATPase-A* synthesis | **F: TAATACGACTCACTATAGGGTTTACCCAGAATCCGTGAT** |
|  |  | **R: TAATACGACTCACTATAGGGATGGAGTGAAGTCCCAAGC** |
| *AlucV-ATPase-D* | primers for EST cloning | **F: CAGTCAACGAAGAAGGGAG** |
|  |  | **R: CAAGGCACTGATTATGGGAG** |
|  | Primers for RT-qPCR | **F: TGCGACAGACGGACTACCTCA** |
|  |  | **R: CTCCCATAATCAGTGCCTTGC** |
|  | Primers for ds*AlucV-ATPase-D* synthesis | **F: TAATACGACTCACTATAGGGGCAGTCAACGAAGAAGGGAG** |
|  |  | **R: TAATACGACTCACTATAGGGCAAGGCACTGATTATGGGAG** |
| *AlucV-ATPase-E* | primers for EST cloning | **F: GAGACAAGGACCAAGACC** |
|  |  | **R: CAAAGCAAGAGTTGAGGG** |
|  | Primers for RT-qPCR | **F: CGGCGGTATCGAACTCCTAG** |
|  |  | **R: CATCGAATTTGCGGTTGATGT** |
|  | Primers for ds*AlucV-ATPase-E* synthesis | **F: TAATACGACTCACTATAGGGGAGACAAGGACCAAGACC** |
|  |  | **R: TAATACGACTCACTATAGGGCAAAGCAAGAGTTGAGGG** |
| *AlucEif-5A* | primers for EST cloning | **F: CACGCTAAAGTTCACTTGGTC** |
|  |  | **R: TTATTTGTCAAGGGCGGTGTT** |
|  | Primers for RT-qPCR | **F: CAAATCATGTGGCGAGGAGTG** |
|  |  | **R: GGGTGGTCGTGTTATTGTGGTT** |
|  | Primers for ds*AlucEIF-5A* synthesis | **F: TAATACGACTCACTATAGGGCACGCTAAAGTTCACTTGGTC** |
|  |  | **R: TAATACGACTCACTATAGGGTTATTTGTCAAGGGCGGTGTT** |
| *AlucIAP* | primers for EST cloning | **F: AAACGGAATCGCAAAGACC** |
|  |  | **R: GTTTCCATTTTGGGTTACATCC** |
|  | Primers for RT-qPCR | **F: CTTAGCGATGCCGGATTCTT** |
|  |  | **R: ACCCAGGGCTCGTCTGTCTC** |
|  | Primers for ds*AlucIAP* synthesis | **F: TAATACGACTCACTATAGGGAAACGGAATCGCAAAGACC** |
|  |  | **R: TAATACGACTCACTATAGGGGTTTCCATTTTGGGTTACATCC** |
| *AlucEcR-A* | primers for EST cloning | **F: CTCGGCAACCCAATCTCC** |
|  |  | **R: AAGGCTTACTGCATTGTGAAC** |
|  | Primers for RT-qPCR | **F: GGGCTGCTGGTGTCAGGAAT** |
|  |  | **R: CCATGTGAGTGAGAAGAGGAGGTT** |
|  | Primers for ds*AlucEcR-A* synthesis | **F: TAATACGACTCACTATAGGGCTCGGCAACCCAATCTCC** |
|  |  | **R: TAATACGACTCACTATAGGGAAGGCTTACTGCATTGTGAAC** |
| *ZmGADPH* | Primers for RT-qPCR | **F: CCCTTCATCACCACGGACTAC** |
|  |  | **R: AACCTTCTTGGCACCACCCT** |
| *GmGADPH* | Primers for RT-qPCR | **F: CGGTGCTGCTAGAGGATGG** |
|  |  | **R: CCCAGAAGCGCCAAGCACA** |
| *GFP* | Primers for ds*GFP* synthesis | **F:TAATACGACTCACTATAGGGGTGGAGAGGGTGAAGGTGAT** |
|  |  | **R:TAATACGACTCACTATAGGGGTGTCCAAGAATGTTTCCATC** |
| *AlucGADPH* | primers for EST cloning | **F: GGATT TGG(TC)CG(TC)AT(TC)GG(CT)C** |
|  |  | **R: GAGTA (AG)CC(AG)(AT)A(TC)TC(AG)TTGTCGTACC** |
|  | Primers for RT-qPCR | **F:TTCCGAGTTCCTGTCCCTAATG** |
|  |  | **R: GCCTCCTTCACCTTCTGCTTG** |
