# Supplementary material for: Plant-Mediated RNAi for Controlling Apolygus lucorum
Source: Front Plant Sci. 2019 Feb 6;10:64. doi: 10.3389/fpls.2019.00064 (PMC6374644; doi:10.3389/fpls.2019.00064)

**Supplementary Data1**

**The alignments of target genes.**

Sequences comparisons were performed using DNAman software. [Target](https://cn.bing.com/dict/clientsearch?mkt=zh-CN&setLang=zh&form=BDVEHC&ClientVer=BDDTV3.5.1.4320&q=%E9%9D%B6%E6%A0%87%E5%9F%BA%E5%9B%A0%E8%B7%9F%E5%8F%8C%E7%BF%85%E7%9B%AE%EF%BC%8C%E9%B3%9E%E7%BF%85%E7%9B%AE%EF%BC%8C%E9%9E%98%E7%BF%85%E7%9B%AE%EF%BC%8C%E8%86%9C%E7%BF%85%E7%9B%AE%E7%9A%84%E4%BB%A3%E8%A1%A8%E7%A7%8D%E5%90%8C%E6%BA%90%E5%9F%BA%E5%9B%A0%E8%BF%9B%E8%A1%8C%E6%AF%94%E5%AF%B9) [genes](https://cn.bing.com/dict/clientsearch?mkt=zh-CN&setLang=zh&form=BDVEHC&ClientVer=BDDTV3.5.1.4320&q=%E9%9D%B6%E6%A0%87%E5%9F%BA%E5%9B%A0%E8%B7%9F%E5%8F%8C%E7%BF%85%E7%9B%AE%EF%BC%8C%E9%B3%9E%E7%BF%85%E7%9B%AE%EF%BC%8C%E9%9E%98%E7%BF%85%E7%9B%AE%EF%BC%8C%E8%86%9C%E7%BF%85%E7%9B%AE%E7%9A%84%E4%BB%A3%E8%A1%A8%E7%A7%8D%E5%90%8C%E6%BA%90%E5%9F%BA%E5%9B%A0%E8%BF%9B%E8%A1%8C%E6%AF%94%E5%AF%B9) were alignment [with](https://cn.bing.com/dict/clientsearch?mkt=zh-CN&setLang=zh&form=BDVEHC&ClientVer=BDDTV3.5.1.4320&q=%E9%9D%B6%E6%A0%87%E5%9F%BA%E5%9B%A0%E8%B7%9F%E5%8F%8C%E7%BF%85%E7%9B%AE%EF%BC%8C%E9%B3%9E%E7%BF%85%E7%9B%AE%EF%BC%8C%E9%9E%98%E7%BF%85%E7%9B%AE%EF%BC%8C%E8%86%9C%E7%BF%85%E7%9B%AE%E7%9A%84%E4%BB%A3%E8%A1%A8%E7%A7%8D%E5%90%8C%E6%BA%90%E5%9F%BA%E5%9B%A0%E8%BF%9B%E8%A1%8C%E6%AF%94%E5%AF%B9) the homologous gene from the [representatives](https://cn.bing.com/dict/clientsearch?mkt=zh-CN&setLang=zh&form=BDVEHC&ClientVer=BDDTV3.5.1.4320&q=%E9%9D%B6%E6%A0%87%E5%9F%BA%E5%9B%A0%E8%B7%9F%E5%8F%8C%E7%BF%85%E7%9B%AE%EF%BC%8C%E9%B3%9E%E7%BF%85%E7%9B%AE%EF%BC%8C%E9%9E%98%E7%BF%85%E7%9B%AE%EF%BC%8C%E8%86%9C%E7%BF%85%E7%9B%AE%E7%9A%84%E4%BB%A3%E8%A1%A8%E7%A7%8D%E5%90%8C%E6%BA%90%E5%9F%BA%E5%9B%A0%E8%BF%9B%E8%A1%8C%E6%AF%94%E5%AF%B9) species of [Diptera](https://cn.bing.com/dict/clientsearch?mkt=zh-CN&setLang=zh&form=BDVEHC&ClientVer=BDDTV3.5.1.4320&q=%E9%9D%B6%E6%A0%87%E5%9F%BA%E5%9B%A0%E8%B7%9F%E5%8F%8C%E7%BF%85%E7%9B%AE%EF%BC%8C%E9%B3%9E%E7%BF%85%E7%9B%AE%EF%BC%8C%E9%9E%98%E7%BF%85%E7%9B%AE%EF%BC%8C%E8%86%9C%E7%BF%85%E7%9B%AE%E7%9A%84%E4%BB%A3%E8%A1%A8%E7%A7%8D%E5%90%8C%E6%BA%90%E5%9F%BA%E5%9B%A0%E8%BF%9B%E8%A1%8C%E6%AF%94%E5%AF%B9) (*Drosophila melanogaster*)[,](https://cn.bing.com/dict/clientsearch?mkt=zh-CN&setLang=zh&form=BDVEHC&ClientVer=BDDTV3.5.1.4320&q=%E9%9D%B6%E6%A0%87%E5%9F%BA%E5%9B%A0%E8%B7%9F%E5%8F%8C%E7%BF%85%E7%9B%AE%EF%BC%8C%E9%B3%9E%E7%BF%85%E7%9B%AE%EF%BC%8C%E9%9E%98%E7%BF%85%E7%9B%AE%EF%BC%8C%E8%86%9C%E7%BF%85%E7%9B%AE%E7%9A%84%E4%BB%A3%E8%A1%A8%E7%A7%8D%E5%90%8C%E6%BA%90%E5%9F%BA%E5%9B%A0%E8%BF%9B%E8%A1%8C%E6%AF%94%E5%AF%B9) [Lepidoptera](https://cn.bing.com/dict/clientsearch?mkt=zh-CN&setLang=zh&form=BDVEHC&ClientVer=BDDTV3.5.1.4320&q=%E9%9D%B6%E6%A0%87%E5%9F%BA%E5%9B%A0%E8%B7%9F%E5%8F%8C%E7%BF%85%E7%9B%AE%EF%BC%8C%E9%B3%9E%E7%BF%85%E7%9B%AE%EF%BC%8C%E9%9E%98%E7%BF%85%E7%9B%AE%EF%BC%8C%E8%86%9C%E7%BF%85%E7%9B%AE%E7%9A%84%E4%BB%A3%E8%A1%A8%E7%A7%8D%E5%90%8C%E6%BA%90%E5%9F%BA%E5%9B%A0%E8%BF%9B%E8%A1%8C%E6%AF%94%E5%AF%B9) (*Helicoverpa armigera*)[,](https://cn.bing.com/dict/clientsearch?mkt=zh-CN&setLang=zh&form=BDVEHC&ClientVer=BDDTV3.5.1.4320&q=%E9%9D%B6%E6%A0%87%E5%9F%BA%E5%9B%A0%E8%B7%9F%E5%8F%8C%E7%BF%85%E7%9B%AE%EF%BC%8C%E9%B3%9E%E7%BF%85%E7%9B%AE%EF%BC%8C%E9%9E%98%E7%BF%85%E7%9B%AE%EF%BC%8C%E8%86%9C%E7%BF%85%E7%9B%AE%E7%9A%84%E4%BB%A3%E8%A1%A8%E7%A7%8D%E5%90%8C%E6%BA%90%E5%9F%BA%E5%9B%A0%E8%BF%9B%E8%A1%8C%E6%AF%94%E5%AF%B9) [Coleoptera](https://cn.bing.com/dict/clientsearch?mkt=zh-CN&setLang=zh&form=BDVEHC&ClientVer=BDDTV3.5.1.4320&q=%E9%9D%B6%E6%A0%87%E5%9F%BA%E5%9B%A0%E8%B7%9F%E5%8F%8C%E7%BF%85%E7%9B%AE%EF%BC%8C%E9%B3%9E%E7%BF%85%E7%9B%AE%EF%BC%8C%E9%9E%98%E7%BF%85%E7%9B%AE%EF%BC%8C%E8%86%9C%E7%BF%85%E7%9B%AE%E7%9A%84%E4%BB%A3%E8%A1%A8%E7%A7%8D%E5%90%8C%E6%BA%90%E5%9F%BA%E5%9B%A0%E8%BF%9B%E8%A1%8C%E6%AF%94%E5%AF%B9)(*Tribolium castaneum*), [Hymenopteran](https://cn.bing.com/dict/clientsearch?mkt=zh-CN&setLang=zh&form=BDVEHC&ClientVer=BDDTV3.5.1.4320&q=%E9%9D%B6%E6%A0%87%E5%9F%BA%E5%9B%A0%E8%B7%9F%E5%8F%8C%E7%BF%85%E7%9B%AE%EF%BC%8C%E9%B3%9E%E7%BF%85%E7%9B%AE%EF%BC%8C%E9%9E%98%E7%BF%85%E7%9B%AE%EF%BC%8C%E8%86%9C%E7%BF%85%E7%9B%AE%E7%9A%84%E4%BB%A3%E8%A1%A8%E7%A7%8D%E5%90%8C%E6%BA%90%E5%9F%BA%E5%9B%A0%E8%BF%9B%E8%A1%8C%E6%AF%94%E5%AF%B9) (*Apis mellifera*), Hemiptera (*Acyrthosiphon pisum*) and the species of maize, soybean and humans. dsRNAs design area marked with blue box.

β-actin


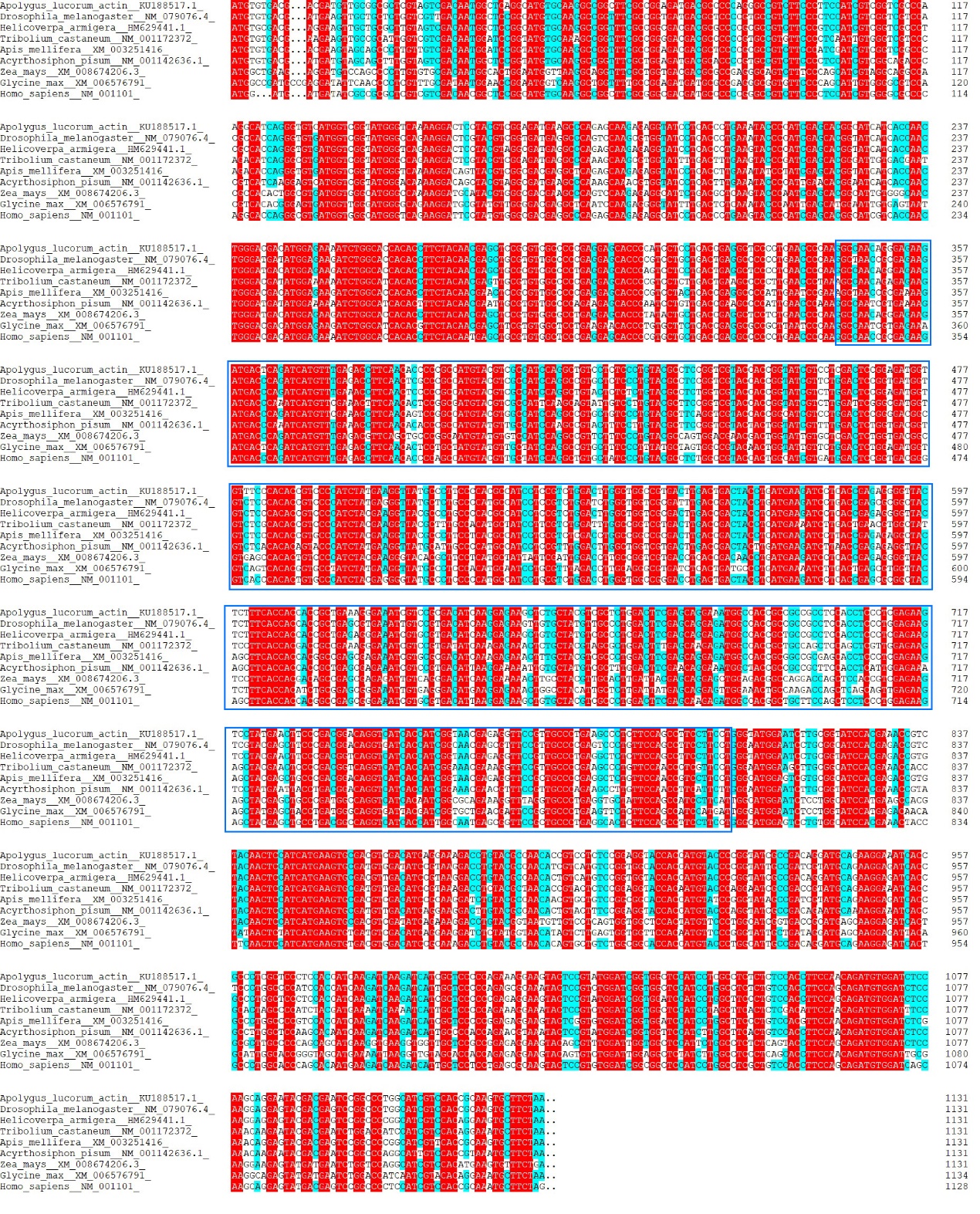


V-ATPase-A


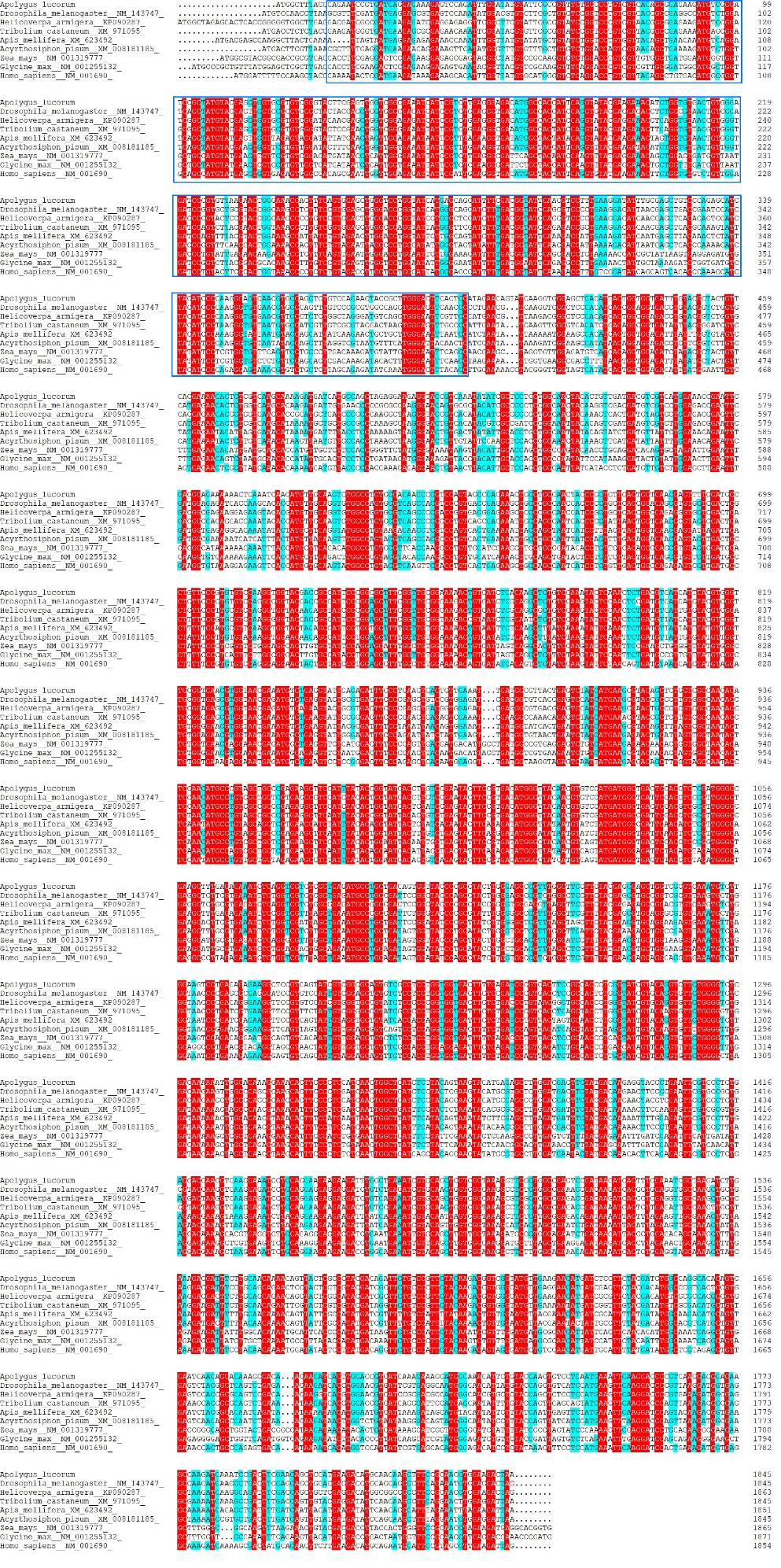


V-ATPase-D


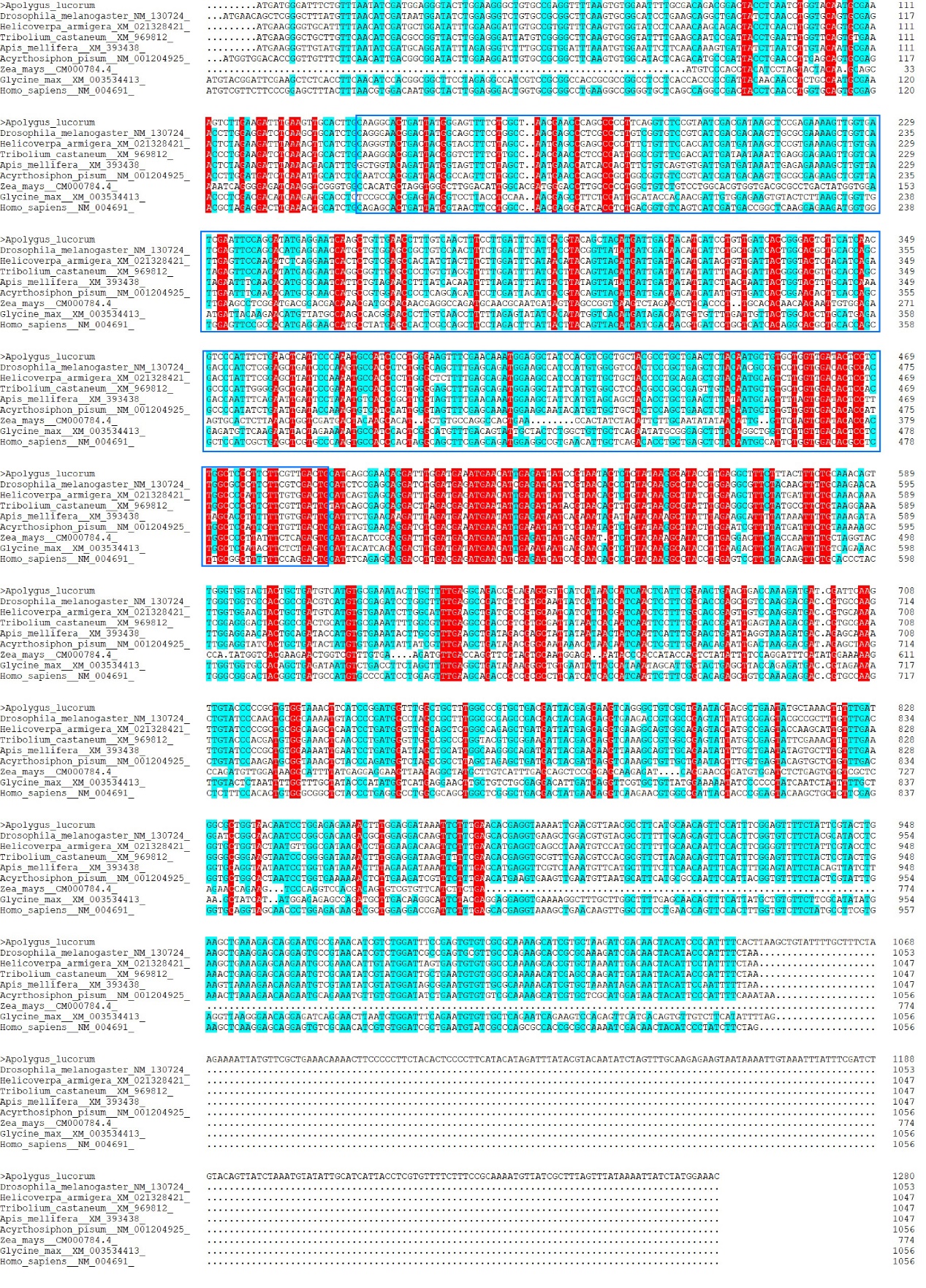


V-ATPase-E


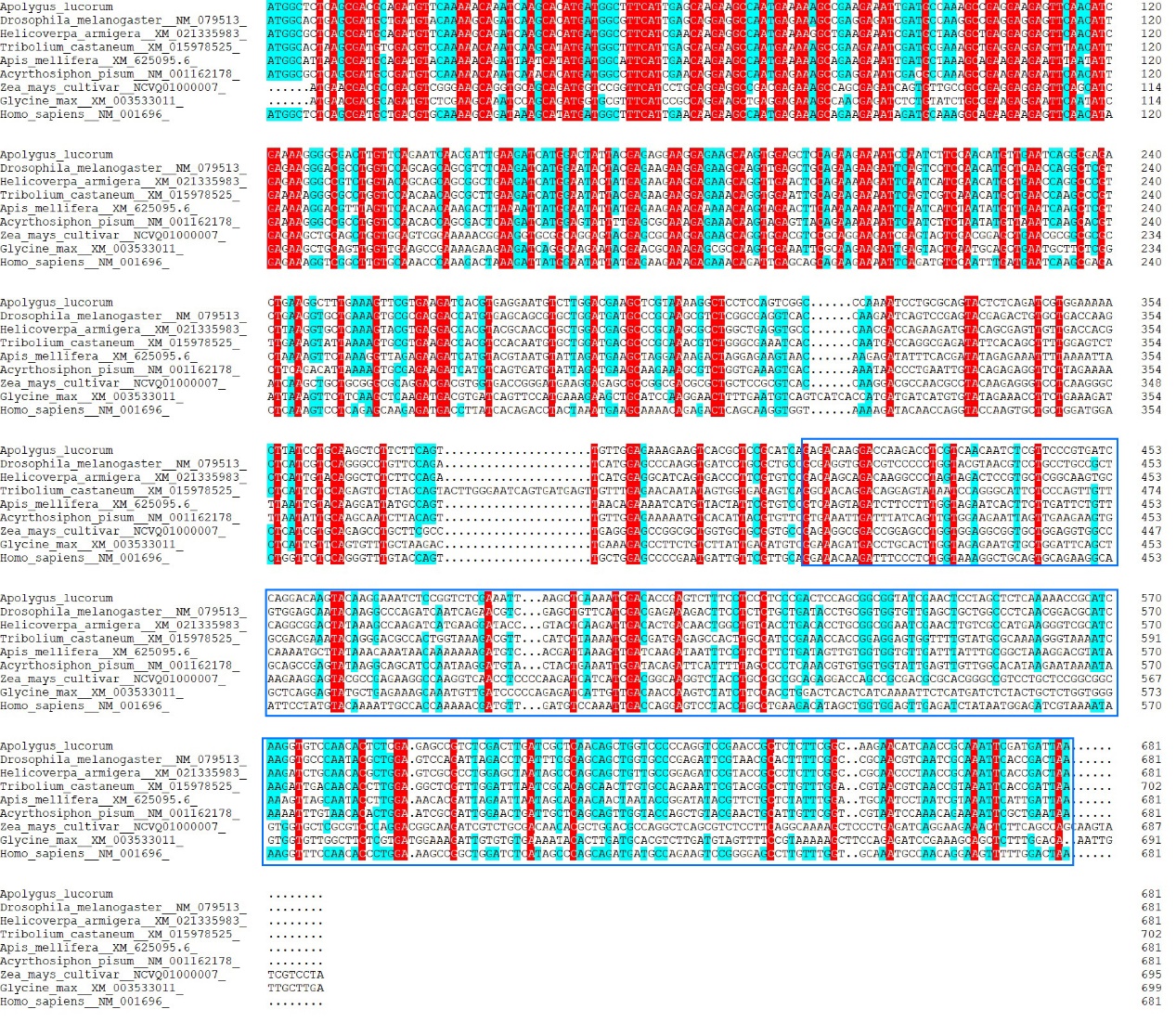


EiF-5A


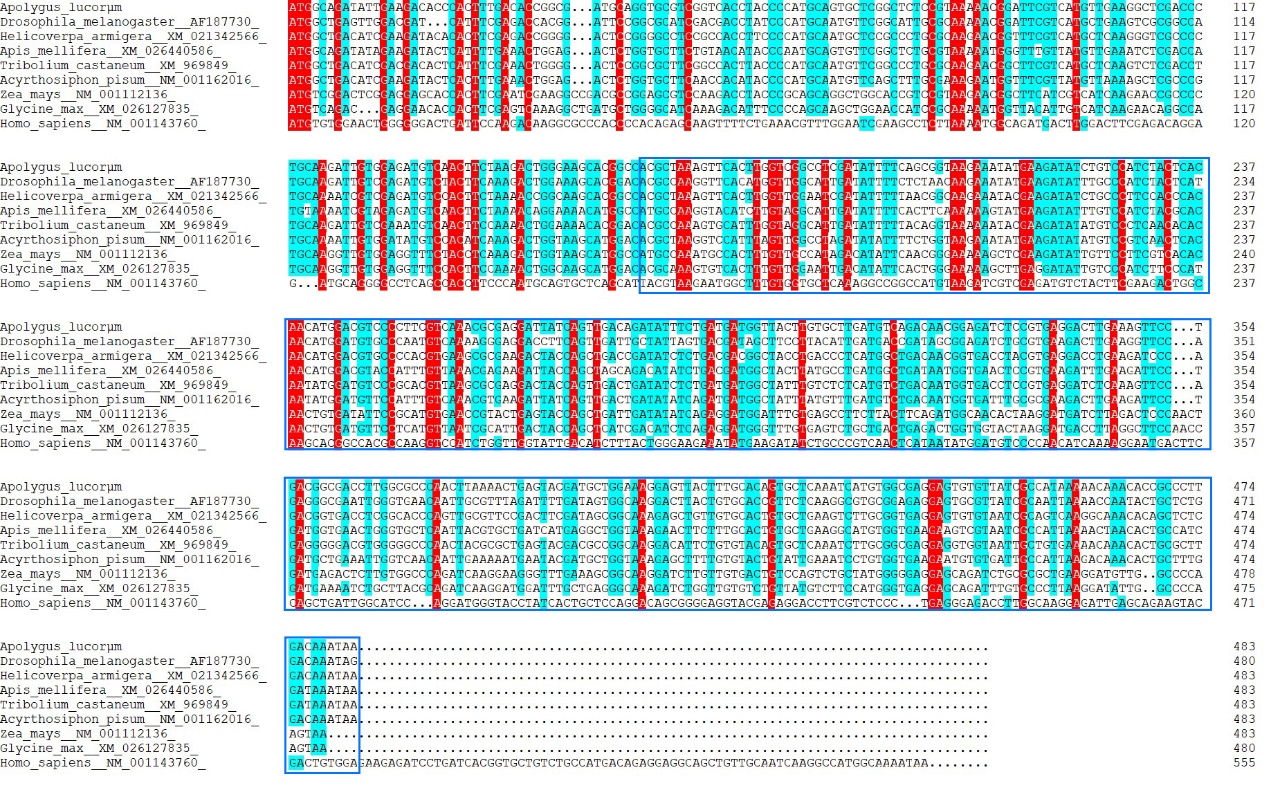


EcR


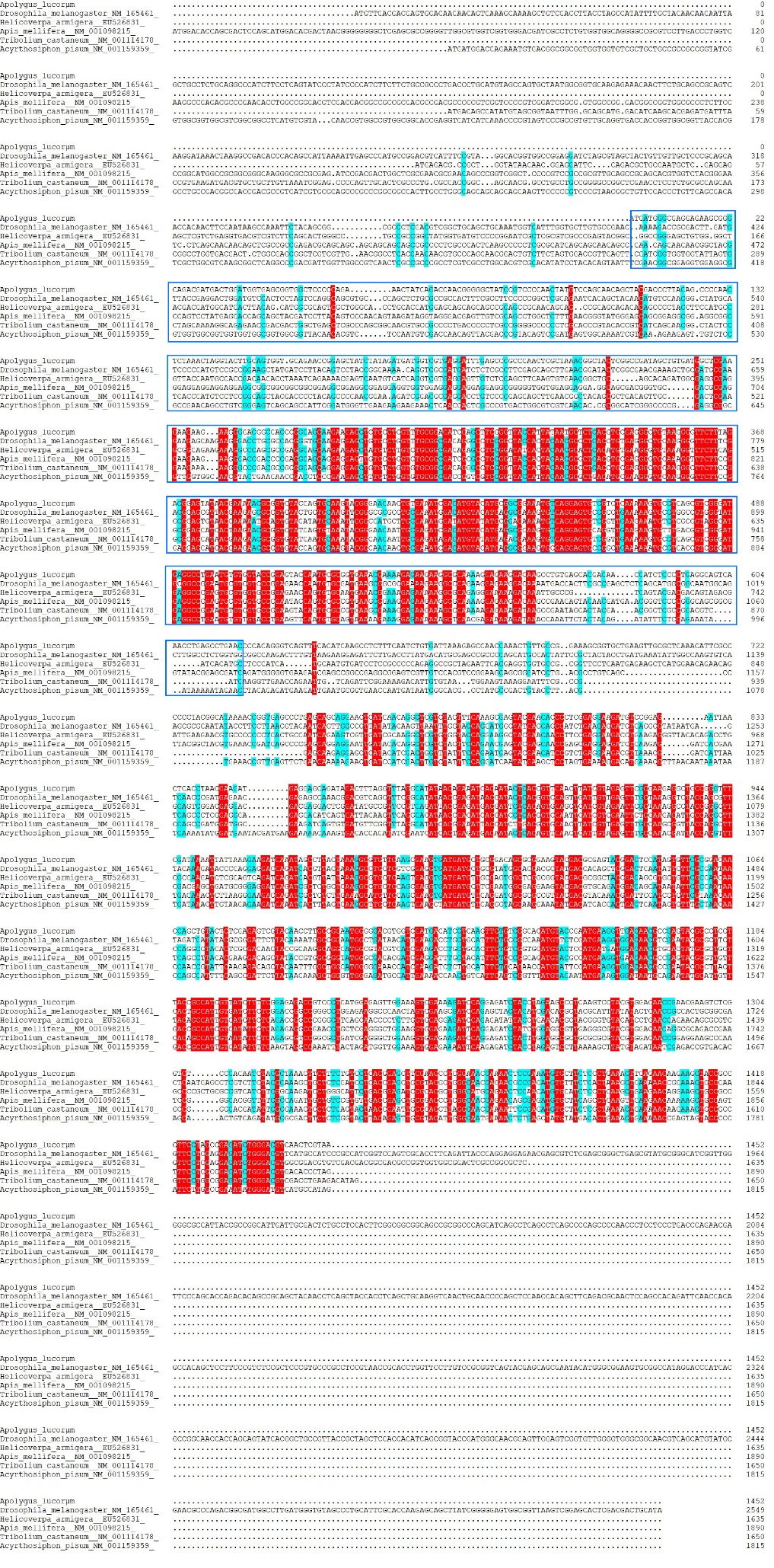


IAP


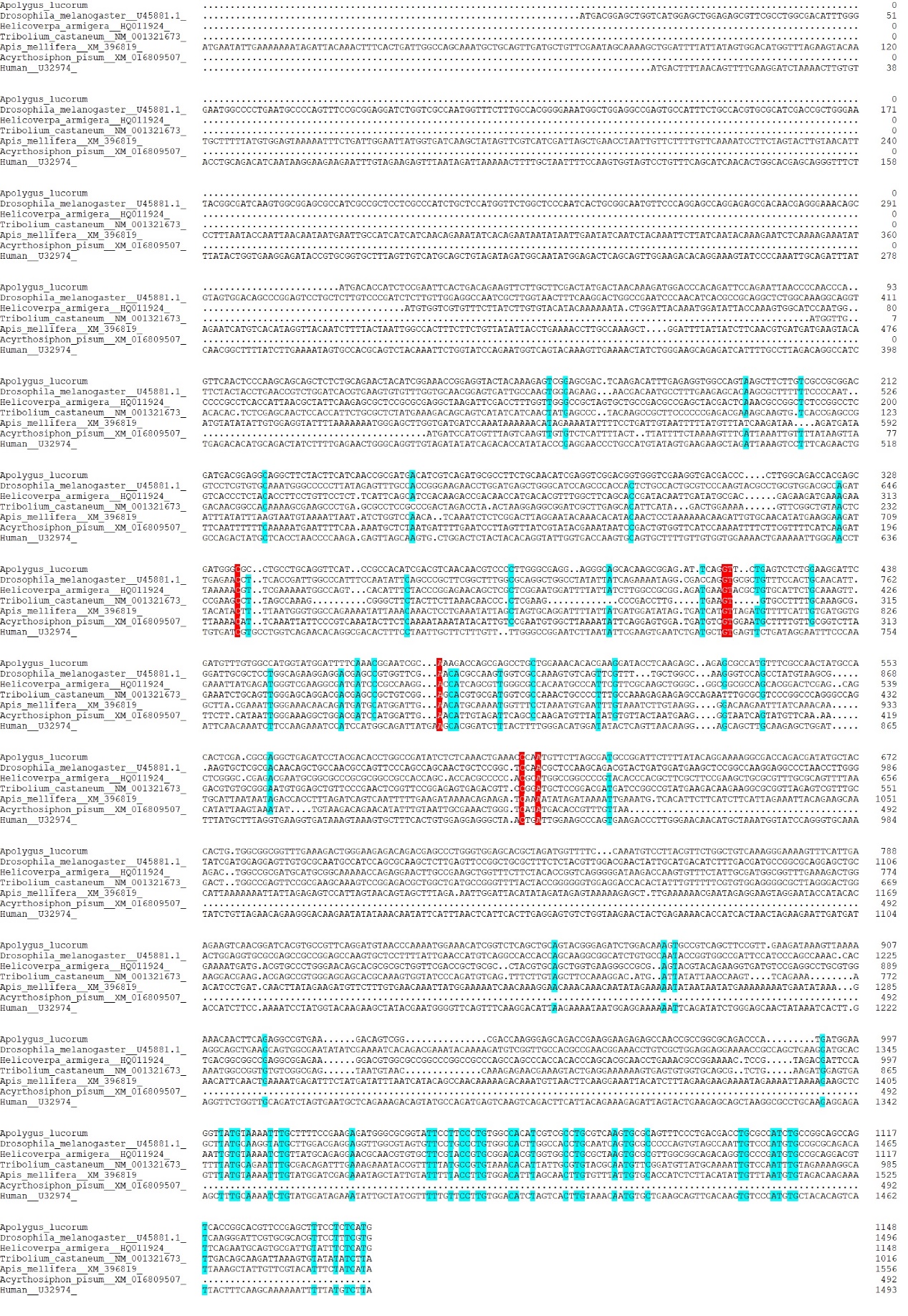

Supplement: Supplementary Data 1 — The alignments of target genes. Sequences comparisons were performed using DNAman software. Target genes were alignment with the homologous gene from the representatives species of Diptera (Drosophila melanogaster), Lepidoptera (Helicoverpa armigera), Coleoptera(Tribolium castaneum), Hymenopteran (Apis mellifera), Hemiptera (Acyrthosiphon pisum) and the species of maize, soybean, and humans. dsRNAs design area marked with blue box. [file Data_Sheet_1.docx]
